# Terry B. Strom: not just a pillar but a foundation

**DOI:** 10.3389/frtra.2024.1519975

**Published:** 2024-12-02

**Authors:** Manikkam Suthanthiran

**Affiliations:** Weill Cornell Medical Center, NewYork-Presbyterian, New York, NY, United States

**Keywords:** transplantation—kidney, immunobiology, pillar, IL-15, lymphocyte-mediated cytotoxicity

## Introduction

**Figure 1:**
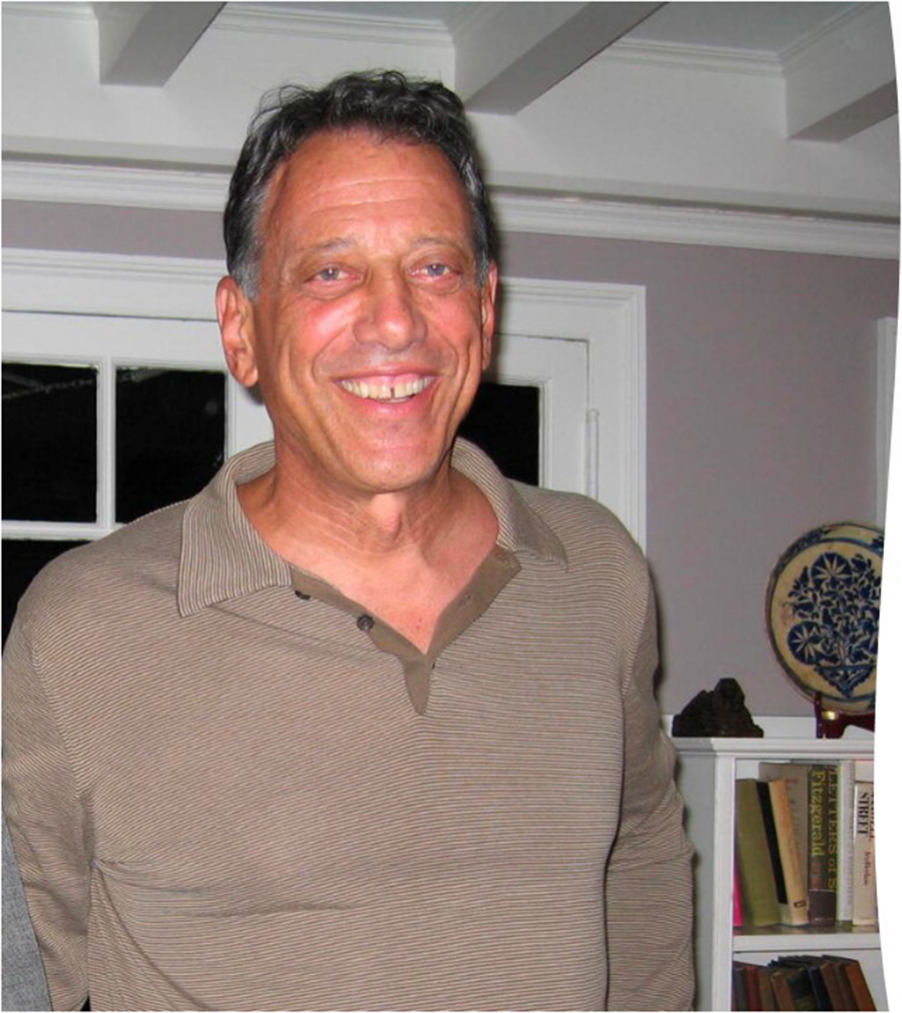
Terry B. Storm, MD. I could have chosen a more formal picture, but I thought this one captures Terry's boyish exuberance and his approach to life: taking the work seriously by not taking himself seriously.

I was delighted when the much-admired Jerzy Kupiec-Weglinski, the erudite editor of Frontiers in Transplantation, invited me to contribute a Pillar article on Terry Barton Strom, MD, the much accomplished and quintessential transplantation immunobiologist of our time (Figure 1).

English being a foreign language for me, I first looked up the meaning of “Pillar” before writing this piece, and I came to the inevitable conclusion that Terry Strom transcended the definition of a pillar and his inspiring and innovative contributions to the transplantation field merited the “Foundation” label as well!

I have also come to realize that there are six degrees of (academic) separation. In the first degree, one is invited to present one's own primary data at a bona fide scientific meeting; in the second, you are asked to summarize your research work in a symposium; in the third degree, one is invited to chair a session on a relevant topic at a meeting but not allowed to speak; in the fourth, one is invited to chair a session at a meeting on a subject one has very little expertise but the invitation was rendered because the invitee occupies an influential position like an Editorship of a Journal or serves on a funding agency; in the fifth degree, one finagles an invitation to participate at an industry-sponsored meeting held at an exotic patch with travel and accommodation far superior to academic societies; in the sixth degree, one's invited to write an obit piece or a pillar article! Of course, writing this article about my inspirational mentor and dear friend, Terry Strom was a labor of love.

An enshrined principle in every religion is that one must leave the world a better place than one found it. Terry Strom most certainly not only enriched the field of immunobiology and transplantation but also stimulated each of us to be better individuals in the highly competitive academic medicine enterprise.

A series of fortunate developments garnered my admission to the Peter Bent Brigham Hospital, Boston, MA in 1975. There was no pampering “orientation” to my fellowship, and I started my transplant service rotation bright and early on July 1, 1975. I had a second lucky break—Terry Strom was my transplant attending! On the first evening of my fellowship, I called Terry to seek his permission to return to Detroit Children's Hospital to attend to my 9-year-old nephew who had just been re-admitted for a post-operative complication following total correction of Tetralogy of Fallot. I was deeply concerned about making this request on my very first day of fellowship. I have not forgotten his most humanistic response that still brings tears to my eyes: “Please go and come back only when you feel comfortable.”

Luck plays a major role in all our lives. I was most fortunate that Terry Strom was not only my brilliant and nurturing mentor but also my most cherished friend. In the laboratory headed by the gentleman-scholar, Charles Bernard Carpenter, Terry Strom provided the spark and served as a stellar role model for taking the science seriously but not himself. His clarity of thought, the complementary eloquence, and the grace with which he treated others were inspirational for me and much admired by one and all. Indeed, at any national or international meeting, if one saw an admiring crowd of scientists in the hallways, Terry Strom was likely to be at the center of the crowd.

My professional career was clearly ignited and sustained by Terry Strom, and he was the “invisible hand” behind my career progression. One of the greatest joys of an investigative career is to collaborate with individuals of high intellect, and it was an unmitigated pleasure to collaborate with Terry who had the rare combination of scientific rigor and generosity of spirit.

## Academic career in brief

**Figure 2:**
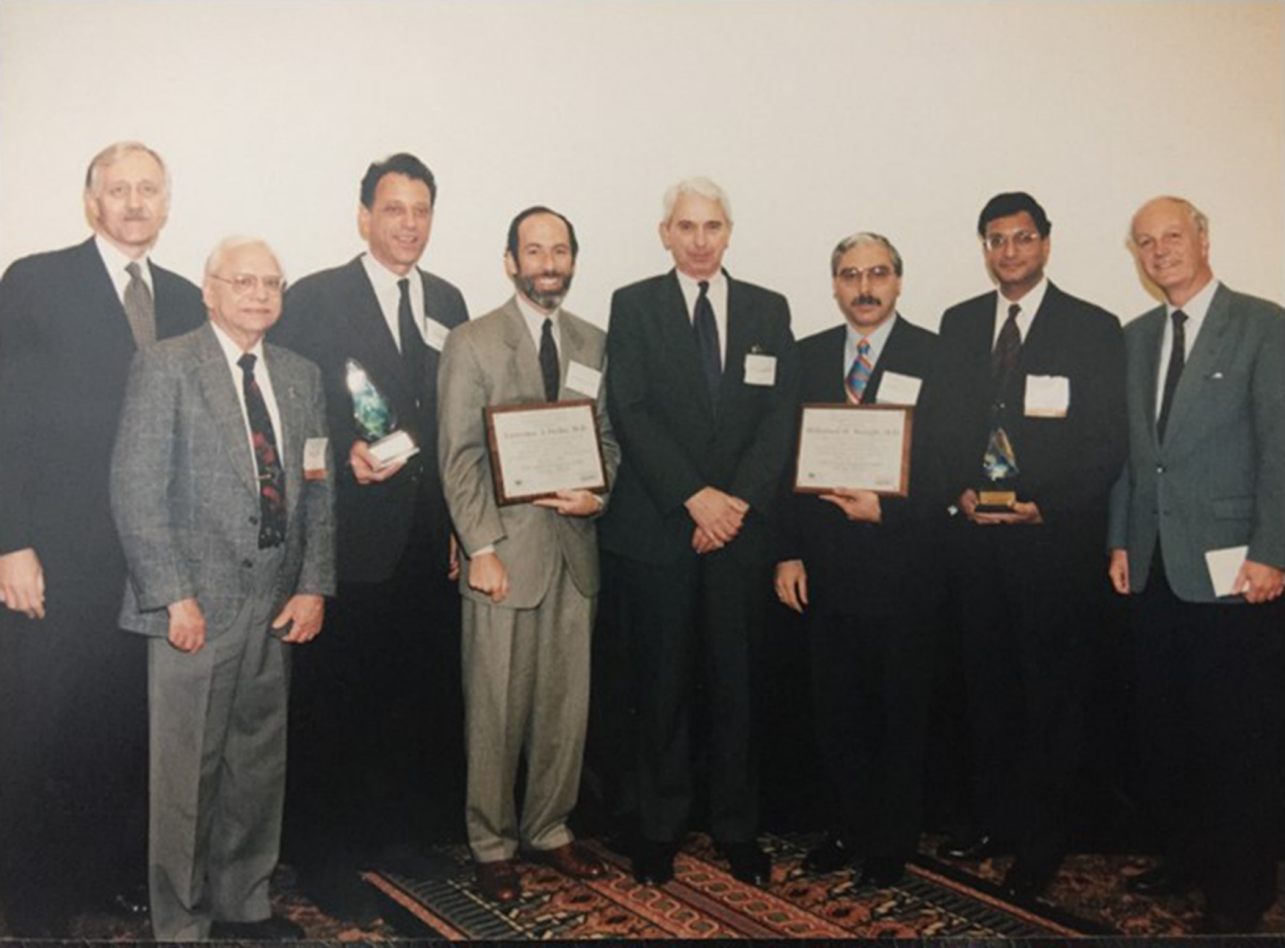
American society of transplantation awardees -circa 1997. L. W. Miller (Pesident, American Society of Transplantation), S.N. Sehgal (Rapamycin pioneer), T.B. Storm, L. A. Turka, C.B. Carpenter, M.H. Sayegh, M. Suthanthiran, J-F. Borel (Cyclosporine pioneer).

Terry Strom's career in transplantation started with his renal fellowship under the legendary John P. Merrill, the Chief of the Cardio-Renal Division at the Peter Bent Brigham Hospital, Boston, MA. Charles (Bernie) Carpenter was the head of the Immunology Laboratory and Terry was the dynamic force in Bernie's laboratory, home to many future practitioners of transplantation immunology. I met Terry in Bernie's lab when he was as a young Assistant Professor of Medicine at the Harvard Medical School. Twenty-two years later, 1991, was a banner year for Bernie's disciples when they garnered all the awards at the annual meeting of the American Society of Transplantation where the awards were presented by luminaries like Suren Sehgal and Jean F Borel (Figure 2). Terry's ascendency on the academic ladder was inevitable and he rose through the ranks to full professorship. In the ledger with loss and gain columns, a significant loss to Brigham and a substantive gain to Beth Israel Hospital was Terry's move to Beth Israel Deaconess Medical Center to become the head of the Department of Immunology with a logistically broadened scope of transplantation and autoimmunity. Terry's magnetic personality attracted an international cadre of talented individuals who have emerged from his inspiring mentorship as leaders of the transplantation immunology field.

## Some of his significant scientific contributions

Terry Strom was an exceptional scientist who defied pigeonholing as a basic, or translational, or an applied researcher. The quantitative aspect of his research productivity was only rivalled by its qualitative features. His original discoveries included the identification of cholinergic agents augmenting lymphocyte-mediated cytotoxicity (1) (LMC), a prototypic model for the effector mechanism contributing to allograft rejection; elucidation of the critical contributions of microtubular assembly to the effector activity in LMC (2); and the discovery of the counter-regulatory role of cyclic nucleotides on LMC (3). Terry and his talented colleagues demonstrated that the hormone insulin, known almost exclusively for its glycemic homeostatic role, enhances cytotoxic activity in the LMC assay (4); that insulin receptors are displayed on alloimmune *T* cells (5); and that the expression of these insulin receptors signal activation status of the two major types on immune cellular elements, the *T* cells and the B cells (6).

The IL-2 receptor alpha (CD25) is essential for the high affinity interaction of the IL-2 receptor complex with its ligand IL-2, and *T* cell clonal expansion. Terry Strom's incisive experiments demonstrating that anti-CD25 monoclonal antibodies prolong the survival of mouse cardiac allografts in fully H-2 mismatched donor-recipient combinations paved the way for the clinical of monoclonal antibodies directed at the CD25 antigen in the clinic for the prevention of allograft rejection (7).

Living up to the Albert Einstein's adage “a man should look for what is, and not for what he thinks should be”, the Strom laboratory unhesitatingly reported the surprising finding that IL-2 knock out mice reject allogeneic islet allografts as vigorously as the wild type mice (8)—a courageous publication in the face of the then prevailing paradigm that IL-2 is essential for allograft rejection. This novel observation also helped also to widen the idea that the salutatory effects of calcineurin inhibitors transcend their ability to block IL-2 transcription. Equally audacious was the publication from the team of Strom Nicholas Tilney that allograft rejection is not only associated with cytotoxic effectors but also with the emergence of suppressor cells (9)—christened as T regulatory cells in the current vernacular.

The IL-2 receptor complex consists of the alpha chain (CD25), beta chain, and common gamma chain. IL-15 has its unique alpha chain but utilizes the same beta chain and gamma chain used by IL-2 for transmembrane signaling of *T* cells. The Strom lab not only introduced IL-15 to the transplant community (10), but successfully deciphered the distinct roles of IL-2 and IL-15 in the destiny of *T* cells (11).

The transformative PCR assay that garnered the Nobel Prize for Kary B. Mullis was brilliantly adopted by the Strom laboratory to profile preclinical (12) and clinical samples (13) and decipher informative intragraft profiles that have endured over time. It was pure joy for my own laboratory to collaborate with Strom's laboratory in broadening the versatile PCR assay to enable absolute quantification of transcripts (14) and advancing urine as a robust surrogate biospecimen for the invasive kidney allograft biopsy (15). Altogether, the Strom laboratory has had a transformative impact on our understanding of allograft rejection and tolerance.

## Peer recognition

Terry Strom's discoveries and paradigm shifting research contributions have been duly recognized by honorific societies. He was elected member of the American Society for Clinical Investigation and the Association of American Physicians. He was the first Past President of the American Society of Transplantation and served as the President of the Clinical Immunology Society. The American Society of Nephrology bestowed its highest honor, the Homer Smith Award, and the University of Pittsburgh recognized Terry with the Starzl Prize in Surgery and Immunology. Terry Strom received every senior award from the American Society of Transplantation: The Established Investigator Award, the Distinguished Achievement award, and the AST Mentoring Award.

## Concluding remarks

The renowned Irish Poet William Butler Yeats closed his memorable 1937 poem “The Municipality Gallery Revisited” closed his memorable piece with this line: “Think where man's glory most begins and ends, And say my glory was I had such friends”. In my case, it was the long and enduring friendship with Terry Strom. An added bonus was his multitalented wife, Dr. Margot Strom.

## Data Availability

The original contributions presented in the study are included in the article/Supplementary Material, further inquiries can be directed to the corresponding author.
